# p53 oncoprotein overexpression correlates with mutagen-induced chromosome fragility in head and neck cancer patients with multiple malignancies.

**DOI:** 10.1038/bjc.1995.194

**Published:** 1995-05

**Authors:** O. Gallo, S. Bianchi, M. L. Giovannucci-Uzzielli, R. Santoro, S. Lenzi, C. Salimbeni, M. Abbruzzese, E. Alajmo

**Affiliations:** Institute of Otolaryngology-Head & Neck Surgery, University of Florence, Italy.

## Abstract

**Images:**


British Journal of Cancer (1995) 71, 1008-1012

© 1995 Stockton Press All rights reserved 0007-0920/95 $12.00

### p53 oncoprotein overexpression correlates with mutagen-induced chromosome fragility in head and neck cancer patients with multiple malignancies

O Gallo[1], S Bianchi[2], ML Giovannucci-Uzzielli[3], R Santoro[1], S Lenzi[3], C Salimbeni[1], M Abbruzzese[3] and E Alajmo[1]

[1]*Institute of Otolaryngology–Head & Neck Surgery,* [2]*Institute of Pathology and* [3]*Department of Pediatrics, Section of Human Genetics, University of Florence, Italy.*

**Summary** In this study, we analysed immunocytochemically p53 expression in first primary and second primary cancers from 25 head and neck cancer patients (HNCPs) with multiple malignancies in comparison with oncoprotein expression in tumour tissues from 25 historical HNCP controls with single cancer in a match-paired analysis. Moreover, we investigated bleomycin-induced chromosome fragility in both groups of HNCPs and in 21 additional healthy controls. Thirty-nine out of 75 tumour specimens analysed (52%) showed positive p53 immunostaining. Eleven out of 25 (44%) from single cancer patients and 28 out of 50 (56%) tumours from HNCPs with multiple malignancies were p53 positive. In the group of multiple primary cancers, nine patients (36%) showed positive staining of both first and second primaries, whereas six (24%) had positive labelling of first primary cancer but not of the subsequent second primary, four (16%) patient showed p53 expression only in the second primary cancer and six (24%) patients showed no p53 immunoreactivity in both tumours. Chromosomal analysis demonstrated a higher sensitivity to clastogens of HNCPs with multiple tumours than of HNCPs with a single cancer ($P<0.01$), and a significant correlation between chromosome fragility and p53 overexpression ($P<0.01$) only in HNCPs with multiple malignancies more than in those with single head and neck cancer ($P=0.11$). Moreover, we found that patients with p53-positive staining of both first and second primaries showed a statistically significant higher mutagen sensitivity than those with a single p53 immunoreactive tumour or those in whom both cancers were p53 negative ($P<0.01$). Our data suggest that subjects with increased susceptibility to carcinogens after exposure to tobacco or alcohol are at higher risk for multiple cancers in which one of the most common genetic events is aberrant p53 expression.

**Keywords**: multiple primary tumours; head and neck cancer; p53 tumour-suppressor gene; chromosome mutagen sensitivity

From 10% to 40% of patients with head and neck cancer are known to have a high risk of developing second primary neoplasms within a few years following initial diagnosis (Shikhani *et al.*, 1989; Larson *et al.*, 1990; Haughey *et al.*, 1992). Factors considered responsible for multiple malignancies in head and neck cancer patients remain incompletely understood.

In agreement with the 'field cancerisation' theory (Slaughter *et al.*, 1953), it is likely that in a fraction of patients with primary cancer of the upper aerodigestive tract prolonged exposure to carcinogens (alcohol, tobacco smoke) could generate genetic changes in the epithelial cells of exposed mucosae, leading to a higher risk of developing second primary malignancies (Franco *et al.*, 1991; Day *et al.*, 1994). The population at highest risk of multiple primary tumours seems to be genetically predisposed, showing an increased susceptibility to carcinogens, as detected by clastogen-induced chromosome fragility within peripheral blood lymphocytes (Schantz *et al.*, 1990).

Molecular genetics has provided evidence that mutations in the p53 tumour-suppressor gene are involved in a large number of primary squamous cell carcinomas of the upper aerodigestive tract (Nigro *et al.*, 1989; Sakai and Tsuchida, 1990; Maestro *et al.*, 1992; Caamano *et al.*, 1993; Field *et al.*, 1993). Moreover, the association between cigarette smoking and p53 gene mutations or overexpression in HNCPs suggests that the p53 tumour-suppressor gene may be a genetic target of environmental carcinogens (Field *et al.*, 1991, 1992). Therefore, it is conceivable that independent somatic mutations of the p53 tumour-suppressor gene in different carcinogen-exposed areas of the upper aerodigestive tract might be key events in the development of multiple malignancies in genetically predisposed HNCPs.

In order to test our hypothesis, we analysed immunocytochemically the expression of p53 oncogene in tumour tissues from HNCPs who developed second primary head and neck cancers in comparison with p53 expression in tumour tissues from historical HNCP controls without multiple malignancies in a matched-pair analysis. Moreover, we attempted to ascertain if p53 oncoprotein overexpression correlates with genetically predisposed high sensitivity to clastogens in patients with multiple malignancies of the upper aerodigestive tract.

### Patients and methods

### *Patients*

Between January 1992 and February 1993 at the Institute of Otolaryngology of the University of Florence, 25 patients with previously treated squamous cell carcinomas of the head and neck who experienced second primaries were selected for our study. The criteria for selection of multiple tumour from recurrent first primary included: (a) different site of tumour growth, and only for second lesions growth at the same primary site; (b) interval between first and second primary longer than 5 years. To compare the patients who developed multiple malignancies with a group of patients with similar prognostic factors who had not developed a second primary during a period similar to the median follow-up of the first group (5.7 years, range 0.6–13), we selected 25 patients to serve as matched controls from a total of 723 patients treated from 1983 to 1986. Using a computerised search system to avoid bias, we selected individual controls with the same factors as the patients with multiple primary cancers without

Correspondence: O Gallo, Institute of Otolaryngology–Head & Neck Surgery, University of Florence, Policlinico di Careggi, V.le Morgagni 85, 50134 Firenze, Italy
Received 9 June 1994; revised 15 December 1994; accepted 15 December 1994.

reference to the clinical outcome, matching each HNCP with multiple tumours to his or her control by several parameters reported in Table I. Tobacco exposure and alcohol consumption were documented retrospectively and recorded in all individuals. For those individuals who smoked cigarettes, pack-year history was calculated by multiplying the number of packs consumed per day by the number of years exposed. Accordingly, patients with a history of more than 30 pack-years and less than 30 pack-years were considered heavy and moderate smokers respectively. Because of limited information only those HNCPs who gave a history of daily consumption of alcohol-containing beverages were considered 'alcohol-exposed'. A third study group, composed of 21 healthy subjects (all over 50 years old and moderate to heavy smokers) were included in the cytogenetic analysis in order to obtain additional comparative data. The sites of first and second primary malignancies are shown in Table II.

*Cytogenetic studies*

Chromosome analyses were performed on the three study groups. The test was performed as described previously (Hsu *et al.*, 1989; Schantz and Hsu, 1989; Spitz *et al.*, 1989), using as mutagen bleomycin sulphate (Sigma, St Louis, MO, USA) at the concentration of 25 $\mu g\ ml^{-1}$. The number of chromatid breaks counted in each culture was finally converted into the number of chromatid breaks per cell (b/c) to facilitate comparison, as described elsewhere (Hsu *et al.*, 1989).

**Table I** Clinical characteristics of head and neck cancer patients with (group I) and without (group II) multiple malignancies

| *Variable* | *Group I* | *Group II* |
|---|---|---|
| Median age (range) (years) | 58 (46–70) | 60 (47–72) |
| Sex | 22 M and 3 F | 22 M and 3 F |
| Smoking history | | |
| Non-smokers | 6 | 6 |
| Moderate smokers | 8 | 9 |
| Heavy smokers | 11 | 10 |
| Alcohol use | 10/25 (40%) | 9/25 (36%) |
| Site of primary | | |
| Larynx | 12 | 12 |
| Oral cavity | 9 | 9 |
| Pharynx | 4 | 4 |
| Total | 25 | 25 |
| Stage[a] | | |
| I | 8 | 7 |
| II | 7 | 6 |
| III | 7 | 8 |
| IV | 3 | 4 |
| Post-operative radiotherapy | 10/25 (40%) | 12/25 (48%) |
| Median follow-up (range) (years) | 8.7 (0.6–13) | 7.2 (4.8–8.2) |
| Average period to develop second primary (range) (years) | 5.7 (0.6–13) | |

[a]Refers to first primary cancer in group I patients.

*Immunocytochemistry*

Formalin-fixed paraffin-embedded specimens of the primary and second primary tumours from each patient were available for immunocytochemical analysis. Immunocytochemistry was performed using murine monoclonal antibody, DO-7, against human p53 protein (Dako, Copenhagen, Denmark), as reported elsewhere (Shin *et al.*, 1994). The staining pattern was assessed by one of us (SB) and classified as (−) for negative or equivocal staining, (+) 1–25% of positive cells, (+ +) 26–50% of positive cells and (+ + +) >50% of positive cells. Only nuclear staining was regarded as specific staining.

*Statistical analysis*

Statistical comparisons were made with the unpaired Student's *t*-test, Fishers's exact test and finally Kruskal–Wallis analysis. The three following indices and their statistical parameters (average variance, standard deviation) were used for chromosomal analysis: %bc, percentage of cells showing at least one breakage; b/c, breaks per cell (average value for each subject); mb/c, breaks per cell (maximum value for each subject). The significance of differences between the mean values of the three groups was evaluated using Student's *t*-test (double-tailed). The same test was applied to assess the significance of association between cigarette smoking (pack-years) and p53 expression.

The significance of the statistical association between the presence of p53 expression and bleomycin-induced chromosome fragility was tested by Fisher's exact test. Within each HNCP group we considered patients to be bleomycin sensitive if they expressed more than 0.2 b/c. In HNCPs with multiple tumours we considered as positive all patients with p53-positive immunostaining in at least one tumour specimen studied.

The analysis of correlations between b/c median values and p53 immunoreactivity in multiple cancers was performed by Kruskal–Wallis analysis. All *P*-values are two-tailed, with values less than 0.05 considered statistically significant.

### Results and discussion

The results of cytogenetic analysis are reported in Tables III–V. The constitutional karyotype appeared normal in all subjects in the three groups involved in our study (Giemsa banding and quinacrine banding). In the untreated cultures (A,B) none of the subjects belonging to the three groups expressed an abnormal frequency of spontaneous chromosome breakage (range 0.01–0.02) (Figure 1). Culture C, treated with bleomycin sulphate, was examined for breakage analysis. There were significant differences in mean values (%bc, b/c, mb/c) between groups I and II, groups I and III, and finally between groups II and III ($P<0.001$). Our data confirm the original reports from Schantz *et al.* (1990), showing increased bleomycin-induced chromosome fragility in HNCPs with multiple malignancies as compared with HNCPs with single cancer in a matched-paired analysis. However, we found b/c values in our patients lower than those reported previously by others (Hsu *et al.*, 1989; Schantz and Hsu, 1989; Spitz *et al.*, 1989).

Seventy-five head and neck squamous cell carcinomas were investigated for elevated levels of p53 gene expression by

**Table II** Second primary site by index tumour in 25 head and neck cancer patients with multiple malignancies (group I)

| *Index tumour* | *Lung* | *Oesophagus* | *Larynx* | *Oral cavity* | *Pharynx* | *Others* |
|---|---|---|---|---|---|---|
| Larynx ($n = 12$) | 1[b] | 1 | 2 | 4 | 4 | – |
| Oral cavity ($n = 9$) | – | 1 | 2 | 3[a] | 2 | 1[b] |
| Oropharynx ($n = 2$) | – | – | – | 2 | – | – |
| Hypopharynx ($n = 2$) | – | – | – | 1 | 1 | – |
| Total ($n = 25$) | 1 | 2 | 4 | 10 | 7 | 1 |

[a]Included one patient with synchronous tumours. [b]Patient with more than two tumours.

immunocytochemistry, of which 50 represented primary and second primary cancers from HNCPs with multiple malignancies and 25 were cancers from historical controls who did not have second primary tumours. The overall rate of positive immunostaining was 52% (39 out of 75); 30.7% (12 out of 39) of these had intense staining, 35.9% (14 out of 39) showed moderate staining, while 33.3% (13 out of 39) were weakly labelled. Moreover, 19 out of 25 (76.0%) patients with multiple malignancies [15 (78.9%) in the first primary], showed positive tumour staining, whereas only 11 out of 25 (44%) primary squamous cell carcinomas from single cancer controls were positive for p53 oncoprotein immunostaining (Tables III and IV). In the group of HNCPs with multiple cancers, nine patients (36%) showed positive staining of both

**Table III** Bleomycin-induced chromosome fragility and p53 expression in head and neck cancer patients with multiple malignancies (group I). Collected data and related descriptive statistics

| Patient | Sex | Cells examined | Cells with breaks (number) | Cells with breaks (%) | Total breaks (number) | Breaks per cell (average) | Breaks per cell (max.) | Smoking history pack/year | Alcohol use | p53 expression First cancer | p53 expression Second cancer |
|---|---|---|---|---|---|---|---|---|---|---|---|
| 93/147 | M | 50 | 20 | 40.00 | 50 | 1.00 | 6 | 55 | + | ++ | +++ |
| 93/148 | F | 50 | 15 | 30.00 | 36 | 0.72 | 4 | 60 | – | + | – |
| 93/149 | M | 36 | 9 | 25.00 | 20 | 0.56 | 5 | 30 | – | ++ | + |
| 93/150 | F | 50 | 14 | 28.00 | 28 | 0.56 | 4 | 55 | + | +++ | ++ |
| 93/151 | M | 25 | 5 | 20.00 | 13 | 0.52 | 5 | – | + | + | – |
| 93/153 | M | 50 | 3 | 6.00 | 3 | 0.06 | 2 | 30 | – | ++ | – |
| 93/154 | M | 38 | 3 | 7.89 | 5 | 0.13 | 2 | – | – | – | – |
| 93/155 | M | 39 | 6 | 15.38 | 9 | 0.23 | 2 | 55 | + | + | – |
| 93/189 | M | 50 | 18 | 36.00 | 36 | 0.72 | 3 | 25 | + | +++ | + |
| 93/190 | M | 45 | 13 | 28.89 | 19 | 0.42 | 3 | – | – | – | – |
| 93/192 | M | 40 | 9 | 22.50 | 13 | 0.33 | 3 | 80 | + | ++ | + |
| 93/226 | M | 50 | 13 | 26.00 | 16 | 0.32 | 2 | 15 | – | – | + |
| 93/230 | M | 50 | 9 | 18.00 | 20 | 0.40 | 4 | 40 | – | +++ | – |
| 93/253 | M | 50 | 8 | 16.00 | 14 | 0.28 | 3 | 30 | + | – | ++ |
| 93/285 | M | 40 | 2 | 5.00 | 3 | 0.08 | 2 | – | – | – | – |
| 93/391 | M | 43 | 6 | 13.95 | 12 | 0.28 | 3 | 25 | + | – | – |
| 93/503 | F | 35 | 5 | 14.29 | 7 | 0.20 | 2 | 35 | – | – | ++ |
| 93/595 | M | 40 | 2 | 5.00 | 3 | 0.08 | 2 | 25 | + | – | – |
| 93/799 | M | 40 | 12 | 30.00 | 15 | 0.38 | 2 | 40 | – | +++ | + |
| 93/828 | M | 30 | 3 | 10.00 | 6 | 0.20 | 3 | – | – | – | ++ |
| 93/850 | M | 40 | 2 | 5.00 | 3 | 0.08 | 2 | 35 | – | – | – |
| 93/877 | M | 50 | 10 | 20.00 | 14 | 0.28 | 3 | 20 | – | +++ | + |
| 93/892 | M | 45 | 11 | 24.44 | 16 | 0.36 | 3 | 90 | – | ++ | +++ |
| 93/913 | M | 30 | 3 | 10.00 | 12 | 0.40 | 1 | 75 | + | +++ | ++ |
| 93/915 | M | 20 | 2 | 10.00 | 5 | 0.25 | 3 | – | – | ++ | – |
| Average | | | | 19.06 | | | 2.96 | | | | |
| Variance | | | | 104.03 | | 0.05 | 1.43 | | | | |
| Standard deviation | | | | 10.20 | | 0.23 | 1.20 | | | | |

**Table IV** Bleomycin-induced chromosome fragility and p53 expression in head and neck cancer patients without multiple malignancies (group II). Collected data and related descriptive statistics

| Patient | Sex | Cells examined | Cells with breaks (number) | Cells with breaks (%) | Total breaks (number) | Breaks per cell (average) | Breaks per cell (max.) | Smoking history pack/year | Alcohol use | p53 expression in primary cancer |
|---|---|---|---|---|---|---|---|---|---|---|
| 93/225 | M | 45 | 7 | 15.56 | 16 | 0.36 | 4 | 50 | + | +++ |
| 93/227 | F | 50 | 1 | 2.00 | 1 | 0.02 | 1 | – | – | – |
| 93/228 | M | 50 | 7 | 14.00 | 16 | 0.32 | 3 | 35 | – | ++ |
| 93/229 | M | 50 | 6 | 12.00 | 12 | 0.24 | 3 | 20 | – | – |
| 93/252 | M | 38 | 3 | 7.86 | 7 | 0.18 | 3 | 30 | – | ++ |
| 93/254 | M | 40 | 6 | 15.00 | 8 | 0.20 | 2 | 55 | + | + |
| 93/255 | M | 45 | 3 | 6.67 | 4 | 0.09 | 2 | – | + | – |
| 93/266 | M | 50 | 5 | 10.00 | 6 | 0.12 | 2 | – | – | – |
| 93/269 | M | 50 | 11 | 22.00 | 13 | 0.26 | 2 | 10 | + | ++ |
| 93/268 | M | 50 | 7 | 14.00 | 11 | 0.22 | 2 | 55 | – | + |
| 93/280 | M | 30 | 3 | 10.00 | 6 | 0.20 | 1 | 25 | – | – |
| 93/281 | M | 28 | 2 | 7.14 | 4 | 0.14 | 2 | 40 | + | – |
| 93/282 | M | 30 | 2 | 6.67 | 3 | 0.10 | 2 | 35 | – | – |
| 93/283 | M | 35 | 4 | 11.43 | 4 | 0.11 | 1 | – | – | – |
| 93/284 | M | 26 | 3 | 11.54 | 8 | 0.31 | 3 | – | – | + |
| 93/502 | F | 30 | 1 | 3.33 | 1 | 0.03 | 1 | 20 | – | – |
| 93/542 | M | 50 | 2 | 4.00 | 2 | 0.04 | 1 | 45 | – | – |
| 93/586 | M | 26 | 2 | 7.69 | 2 | 0.08 | 1 | 25 | – | – |
| 93/796 | M | 50 | 2 | 4.00 | 4 | 0.08 | 2 | 20 | + | – |
| 93/829 | F | 50 | 4 | 8.00 | 9 | 0.18 | 3 | – | – | + |
| 93/798 | M | 50 | 7 | 14.00 | 16 | 0.32 | 3 | 10 | – | – |
| 93/830 | M | 40 | 3 | 7.50 | 5 | 0.13 | 2 | 55 | + | +++ |
| 93/851 | M | 35 | 2 | 5.71 | 2 | 0.06 | 1 | 25 | + | – |
| 93/914 | M | 30 | 1 | 3.33 | 1 | 0.03 | 1 | 90 | + | +++ |
| 93/916 | M | 20 | 1 | 5.00 | 2 | 0.10 | 2 | 60 | – | +++ |
| Average | | | | 9.17 | | 0.16 | 2.04 | | | |
| Variance | | | | 22.32 | | 0.01 | 0.76 | | | |
| Standard deviation | | | | 4.72 | | 0.10 | 0.87 | | | |

**Table V** Bleomycin-induced chromosome fragility in 21 healthy controls (group III). Collected data and related descriptive statistics

| Patient | Sex | Cells examined | Cells with breaks (number) | Cells with breaks (%) | Total breaks (number) | Breaks per cell (average) | Breaks per cell (max.) | Smoking history pack/year |
|---|---|---|---|---|---|---|---|---|
| 93/332 | M | 20 | 0 | 0 | 0 | 0.00 | 0 | 50 |
| 93/333 | F | 25 | 2 | 8 | 2 | 0.08 | 1 | 25 |
| 93/378 | F | 50 | 4 | 8 | 5 | 0.10 | 2 | 35 |
| 93/403 | F | 50 | 4 | 8 | 6 | 0.12 | 2 | 40 |
| 93/376 | F | 40 | 3 | 7.5 | 3 | 0.08 | 1 | 45 |
| 93/338 | M | 12 | 0 | 0 | 0 | 0.00 | 0 | 35 |
| 93/339 | F | 50 | 2 | 4 | 2 | 0.04 | 1 | 55 |
| 93/492 | F | 50 | 3 | 6 | 5 | 0.10 | 2 | 70 |
| 93/493 | M | 20 | 0 | 0 | 0 | 0.00 | 0 | 90 |
| 93/576 | M | 35 | 3 | 8.5 | 3 | 0.09 | 1 | 40 |
| 93/577 | F | 50 | 3 | 6 | 3 | 0.06 | 1 | 30 |
| 93/635 | M | 40 | 2 | 5 | 3 | 0.08 | 2 | 30 |
| 93/647 | M | 50 | 2 | 4 | 2 | 0.04 | 1 | 35 |
| 93/668 | M | 20 | 0 | 0 | 0 | 0.00 | 1 | 55 |
| 93/678 | M | 50 | 2 | 4 | 3 | 0.06 | 2 | 40 |
| 93/788 | M | 50 | 1 | 2 | 1 | 0.02 | 1 | 40 |
| 93/811 | M | 50 | 1 | 2 | 1 | 0.02 | 1 | 30 |
| 93/820 | M | 50 | 0 | 0 | 0 | 0.00 | 0 | 55 |
| 93/833 | M | 45 | 1 | 2.2 | 1 | 0.02 | 1 | 75 |
| 93/842 | M | 45 | 1 | 2.2 | 2 | 0.04 | 2 | 60 |
| 93/850 | M | 50 | 2 | 4 | 2 | 0.04 | 1 | 45 |
| Average | | | | 3.98 | | 0.05 | 1.10 | |
| Variance | | | | 9.40 | | 0.00 | 0.51 | |
| Standard deviation | | | | 3.07 | | 1.04 | 0.71 | |

**Figure 1** Multiple chromatid breaks (arrows) in a lymphocyte from a primary blood culture treated with bleomycin sulphate ($25\,\mu g\,ml^{-1}$) in an HNCP with multiple malignancies.

first and second primary cancers, whereas six (24%) had positive labelling of the first primary cancer but not of the subsequent second primary, four (16%) patients showed no p53 overexpression in the initial primary but did have positive staining in subsequent primary cancers and six patients (24%) showed no p53 immunoreactivity in both tumours (Table III). Therefore, in this group the overall frequencies of p53 overexpression among primary tumours and second primary cancers were 60% (15 out of 25) and 52% (13 out of 25) respectively.

The analysis of smoking history and of p53 expression in the 50 patients examined showed that patients with p53-positive tumours consumed significantly more cigarettes (mean 50.3 ± 7.5 pack-years) than patients with negative p53 tumours (mean 17.8 ± 3.2) ($P<0.001$). In addition, most of the non-smoking HNCPs (four out of six patients, i.e. 66%) in both groups analysed showed no p53 immunostaining in tumour tissues.

The incidence of 52% of p53-positive tumour specimens analysed is consistent with the reported frequencies for head and neck cancer and other solid tumours (Watling *et al*., 1992; Anwar *et al*., 1993; Caamano *et al*., 1993; Harris and Hollstein, 1993). Detailed analysis of results in the two groups of HNCPs analysed shows that the incidence of p53 overexpression in initial primary head and neck cancer is lower in patients with single tumours than in those with multiple malignancies (44% *vs* 60%), and that the p53 tumour-suppressor gene is overexpressed in 52% (13 out of 25) of second primary cancers and in 36% (9 out of 25) of both primary and second primary cancers from HNCPs with multiple malignancies. These data further support the recently reported data (Chung *et al*., 1993; Nees *et al*., 1993) showing discordant p53 gene mutations in primary and corresponding second primary cancers of the upper aerodigestive tract, and in uninvolved tumour-distant epithelia of head and neck cancer patients, suggesting a new possible molecular basis for the development of multiple tumours.

Correlation between mutagen-induced sensitivity, measured as number of chromatid breaks per cell (b/c), and p53 tumour expression was statistically significant in all the HNCPs taken together ($P<0.001$). Detailed analysis of the separate groups showed that a significant correlation between clastogen-induced chromosome fragility and p53 tumour-suppressor gene expression exists only in patients with multiple malignancies ($P<0.01$), but not in HNCPs with a single tumour ($P=0.12$). Moreover, in the group of patients with multiple malignancies a statistically significant correlation exists between chromosome fragility and p53 immunostaining. Patients with two tumours both p53 positive, had higher b/c values than those with a single p53-positive cancer or those with two p53-negative tumours (median b/c value 0.510, 0.306 and 0.178 respectively) ($\chi^2=9.32$, $P<0.02$).

These data could suggest a major role for genetic factors in the development of multiple tumours in HNCPs. In fact, the sensitivity to bleomycin-induced mutagenesis may reflect a defective DNA repair capability in the host with a high susceptibility to the genotoxic effect of environmental carcinogens such as tobacco and alcohol (Rarshad *et al*., 1983; Hsu *et al*., 1989; Schantz and Hsu, 1989). Accordingly, the analysis of p53 oncoprotein expression in our patients confirms the previously reported correlation between p53 gene mutation or overexpression and cigarette smoking in head and neck and lung cancer patients (Field *et al*., 1993; Westra *et al*., 1993). Thus, it is likely that subjects with increased susceptibility to carcinogens after exposure to tobacco or alcohol are at highest risk for multiple cancers in which one of the most common genetic events is aberrant p53 expression.

Recently, it has been reported that mutations in the p53 gene can also reveal an increased susceptibility to cancer owing to inheritance of p53 germline mutations, as frequently occurs in the cancer-prone individuals with Li-Fraumeni syndrome (Harris, 1993). We demonstrate here that an inherited cancer predisposition expressed by mutagen sensitivity correlates with aberrant p53 gene expression. Thus, differences in such genetic susceptibility and in p53 expression in carcinogen-exposed epithelia of the upper aerodigestive tract might have a major impact in head and neck cancer risk assessment.

Furthermore, we detected p53-positive cells in dysplastic and normal mucosa adjacent to tumour cells in five patients with multiple malignancies. Our data and the identification of p53 overexpression in premalignant lesions of the aerodigestive tract by others (Dolcetti *et al*., 1992; Wang *et al*., 1993; Shin *et al*., 1994) suggest that p53 aberrations are an early event in the development of cancers of the upper aerodigestive tract. Thus, according to the field cancerisation theory, prolonged exposure to environmental carcinogens may induce, in different areas, somatically acquired, p53 gene mutations in normal cells with a high risk of developing multiple cancer lesions, particularly in genetically predisposed subjects.

**Acknowledgements**
We are very grateful to Elena Giannelli and Grazia Asirelli for their technical assistance in p53 immunostaining, to Vanessa Cunial for cytogenetic analysis and to Professor Vieri Boddi for statistical analysis.

Preliminary data were presented at the 1st International Conference on 'Multiple Primary Tumors in Head & Neck Cancers', Trieste, Italy, April 1993.

**References**

ANWAR K, NAKAKUKI K, IMAI H, NAIKI H AND INUZUKA M. (1993). Overexpression of p53 protein in human laryngeal carcinoma. *Int. J. Cancer*, **53**, 952-956.

CAAMANO J, ZHANG SY, ROSVOLD EA, BAUER B AND KLEIN-SZANTO AJP. (1993). p53 alterations in human squamous cell carcinomas and carcinoma cell lines. *Am. J. Pathol.*, **142**, 1131-1139.

CHUNG KY, MUKHOPADHYAY T, KIM J, CASSON A, RO JY, GOEPFERT H, HONG WK AND ROTH JA. (1993). Discordant p53 gene mutations in primary head and neck cancers and corresponding second primary cancers of the upper aerodigestive tract. *Cancer Res.*, **53**, 1676-1683.

DAY LG, BLOT WJ, SHORE RE, McLAUGHLIN JK, AUSTIN DF, GREENBERG RS, LIFF JM, PRESTON-MARTIN S, SARKAR S, SHOENBERG JB AND FRAUMENI Jr JF. (1994). Second cancers following oral and pharyngeal cancers: role of tobacco and alcohol. *J. Natl Cancer Inst.*, **86**, 131-137.

DOLCETTI R, DOGLIONI C, MAESTRO R, GASPAROTTO D, BARZAN L, PASTORE A, ROMANELLI M AND BAIOCCHI M. (1992). p53 over-expression is an early event in the development of human squamous-cell carcinoma of the larynx: genetic and prognostic implications. *Int. J. Cancer*, **52**, 178-182.

FIELD JK, SPANDIDOS DA, MALLIRI A, GOSNEY JR, YIAGNISIS M AND STELL PM. (1991). Elevated p53 expression correlates with a history of heavy smoking in squamous cell carcinoma of the head and neck. *Br. J. Cancer*, **64**, 573-577.

FIELD JK, SPANDIDOS D AND STELL PM. (1992). Overexpression of the p53 gene in head and neck cancer, linked with heavy smoking and drinking. *Lancet*, **339**, 502-503.

FIELD JK, PAVELIC ZP, SPANDIDOS DA, STAMBROOK PJ, JONES AS AND GLUCKMAN JL. (1993). The role of the p53 tumor suppressor gene in squamous cell carcinoma of the head and neck. *Arch. Otolaryngol. Head Neck Surg.*, **119**, 1118-1122.

FRANCO EL, KOWALSJI LP AND KANDA JL. (1991). Risk factor for second cancers of the upper respiratory and digestive system: a case control study. *J. Clin. Epidemiol.*, **44**, 615-625.

HARRIS CC. (1993). p53: at the crossroads of molecular carcinogenesis and risk assessment. *Science*, **262**, 1980-1981.

HARRIS CC AND HOLLSTEIN M. (1993). Clinical implications of the p53 tumor-suppressor gene. *New Engl J. Med.*, **329**, 1318-1327.

HAUGHEY BH, GATES AG, ARFKEN CL AND HARVEY J. (1992). Meta-analysis of second malignant tumors in head and neck cancer: the case for an endoscopic screening protocol. *Ann. Otol. Rhinol. Laryngol.*, **101**, 105-112.

HOLLSTEIN M, SINDRASKY D, VOLGESTEIN B AND HARRIS CC. (1991). p53 mutations in human cancers. *Science*, **253**, 49-53.

HSU TC, JOHNSTON DA, CHERRY LM, RAMKISSOON D, SCHANTZ SP, JESSUP JM, WINN RJ, SHIRLEY L AND FURLONG C. (1989). Sensitivity to genotoxic effects of bleomycin in humans: possible relationship to environmental carcinogenesis. *Int. J. Canceer*, **43**, 403-409.

LARSON TJ, ADAMS GL AND FATAH HA. (1990). Survival statistics for multiple primaries in head and neck cancer. *Otolaryngol. Head Neck Surg.*, **103**, 14-24.

LEVINE AJ. (1992). The p53 tumor-suppressor gene. *New Engl J. Med.*, **326**, 1350-1351.

MAESTRO R, DOLCETTI R, GASPAROTTO D, DOGLIONI C, PELUCCHI S, BARZAN L, GRANDI E AND BOIOCCHI M. (1992). High frequency of p53-gene alteration associated with protein overexpression in human squamous-cell carcinoma of the larynx. *Oncogene*, **7**, 1159-1166.

NIGRO JM, BAKER SJ, PREISINGER AC, JESSUP JM, HOSTETTER R, CLEARY K, BIGNER SH, DAVID N, BAYLIN S, DEVILEE P, GLOVER T, COLLINS FS, WESTON A, MODALI R, HARRIS CC AND VOLGESTEIN B. (1989). Mutations in p53 gene occur in diverse human tumor types. *Nature*, **342**, 705-708.

NEES M, HOMANN N, DISCHER H, ANDL T, ENDERS C, HEROLD-MENDE C, SCHUHMANN A AND BOSCH FX. (1993). Expression of mutated p53 occurs in tumor-distant epithelia of head and neck cancer patients: a possible molecular basis for the development of multiple tumors. *Cancer Res.*, **53**, 4189-4196.

PARSHAD R, SANFORD KK AND JONES GM. (1993). Chromatid damage after $G_2$ phase X-irradiation of cells from cancer-prone individuals implicates deficiency in DNA repair. *Proc. Natl Acad. Sci. USA*, **80**, 5612-5616.

SAKAI E AND TSUCHIDA N. (1992). Most human squamous cell carcinomas in the oral cavity contain mutated p53 tumor suppressor-gene. *Oncogene*, **7**, 927-933.

SCHANTZ SP AND HSU TC. (1989). Mutagen-induced chromosome fragility within peripheral blood lymphocytes of head and neck cancer patients. *Head & Neck*, **11**, 337-342.

SCHANTZ SP, SPITZ MR AND HSU TC. (1990). Mutagen sensitivity of patients with head and neck cancers: a biologic marker for risk of multiple primary malignancies. *J. Natl Cancer Inst.*, **82**, 1773-1775.

SHIN DM, KIM J, RO YJ, HITTELMAN J, ROTH JA, HONG WK AND HITTELMAN WN. (1994). Activation of p53 gene expression in premalignant lesions during head and neck tumorigenesis. *Cancer Res.*, **54**, 321-326.

SHIKHANI AH, MATANOSKI GM, JONE MM, KASHIMA HK AND JOHNS ME. (1989). Multiple primary malignancies in head and neck cancer. *Arch. Otolaryngol. Head Neck Surg.*, **112**, 1172-1179.

SLAUGHTER DP, SOUTHWICH H AND SMEJKAL W. (1953). 'Field cancerization' in oral stratified squamous epithelium. *Cancer*, **6**, 963-968.

SPITZ MR, FUEGER JJ, BENNINGFIELD NA, ANNEGERS JF, HSU TC, NEWELL GR AND SCHANTZ SP. (1989). Chromosome sensitivity to bleomycin-induced mutagenesis, an independent risk factor for upper aerodigestive tract cancers. *Cancer Res.*, **49**, 4626-4628.

VOLGESTEIN B AND KINZIER KW. (1992). p53 function and dysfunction. *Cell*, **70**, 523-552.

WANG L, HONG J, QIU S, GAO H AND YANG CS. (1993). Accumulation of p53 protein in human esophageal precancerous lesions: a possible early biomarker for carcinogenesis. *Cancer Res.*, **53**, 1783-1787.

WATLING DL, GOWN AM AND COLTRERA MD. (1992). Overexpression of p53 in head and neck cancer. *Head & Neck*, **14**, 437-444.

WESTRA WH, OFFERHAUS JA, GOODMAN SN, POLAK M, BAAS IO, RODENHUIS S AND HRUBAN RH. (1993). Overexpression of the p53 tumor suppressor gene product in primary lung adenocarcinomas is associated with cigarette smoking. *Am. J. Surg. Pathol.*, **17**, 213-220.


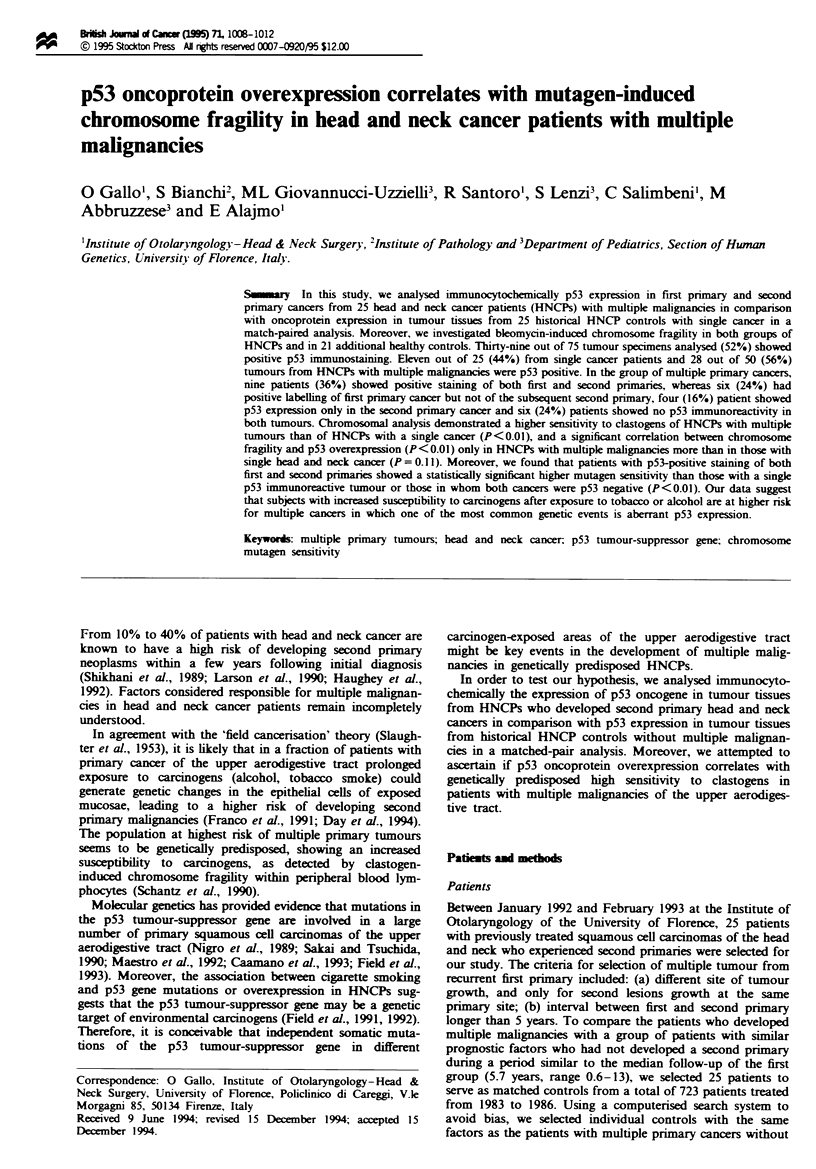

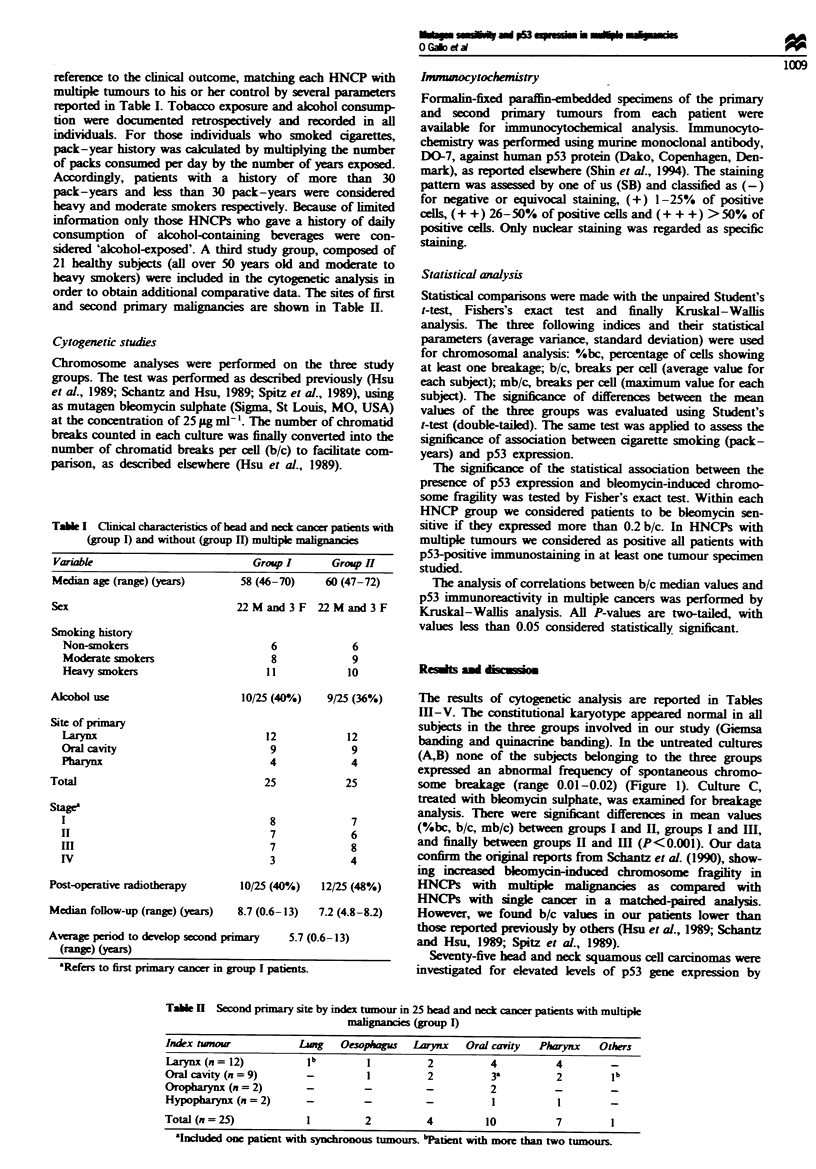

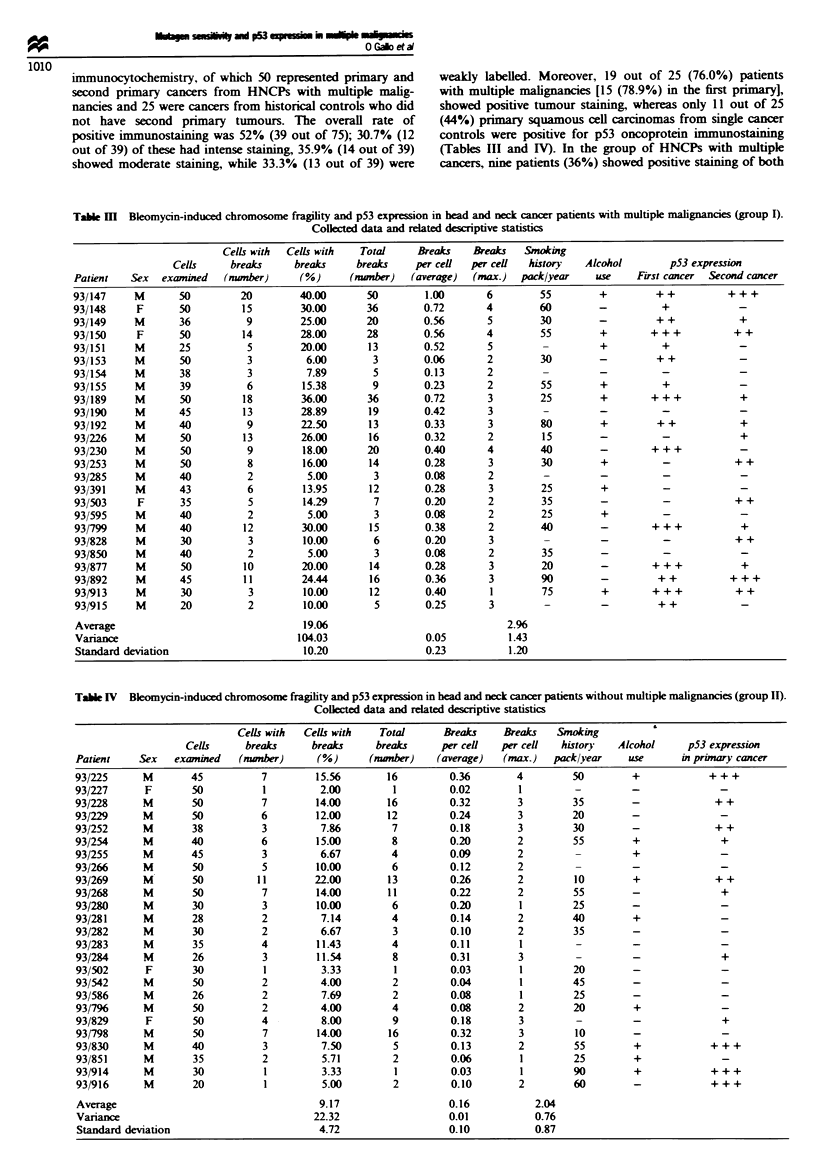

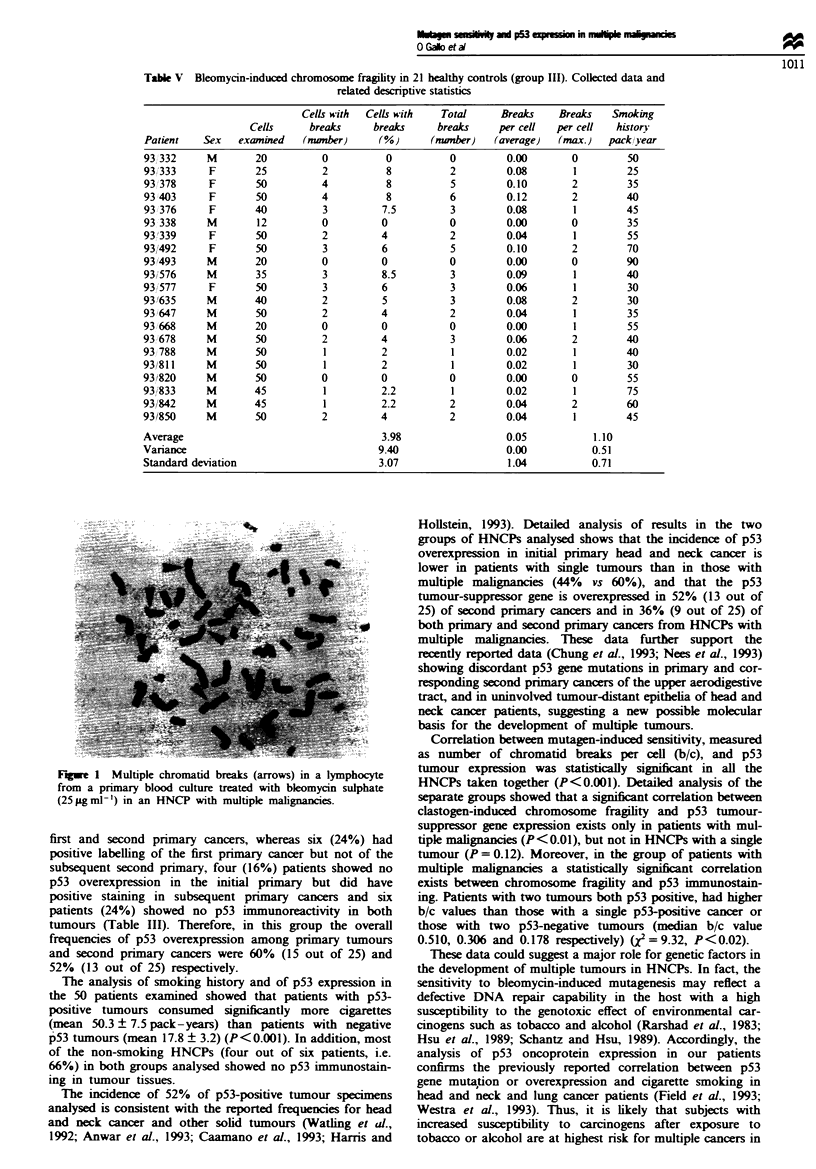

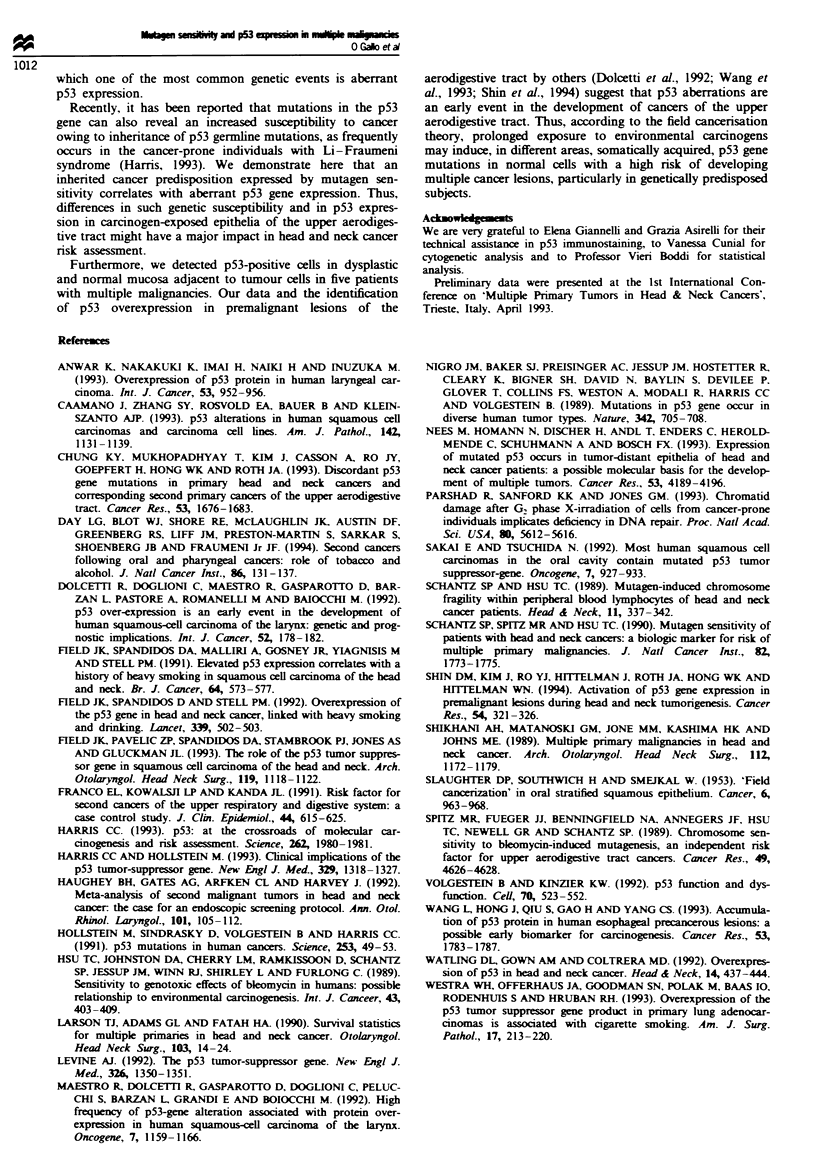


## References

[OCR_00004] Abe F, Albrow MG, Amendolia SR, Amidei D, Antos J, Anway-Wiese C, Apollinari G, Areti H, Atac M, Auchincloss P (1995). Search for second generation leptoquarks in pp-bar collisions at sqrt s=1.8 TeV.. Phys Rev Lett.

[OCR_00807] Anwar K., Nakakuki K., Imai H., Naiki H., Inuzuka M. (1993). Over-expression of p53 protein in human laryngeal carcinoma.. Int J Cancer.

[OCR_00813] Caamano J., Zhang S. Y., Rosvold E. A., Bauer B., Klein-Szanto A. J. (1993). p53 alterations in human squamous cell carcinomas and carcinoma cell lines.. Am J Pathol.

[OCR_00816] Chung K. Y., Mukhopadhyay T., Kim J., Casson A., Ro J. Y., Goepfert H., Hong W. K., Roth J. A. (1993). Discordant p53 gene mutations in primary head and neck cancers and corresponding second primary cancers of the upper aerodigestive tract.. Cancer Res.

[OCR_00823] Day G. L., Blot W. J., Shore R. E., McLaughlin J. K., Austin D. F., Greenberg R. S., Liff J. M., Preston-Martin S., Sarkar S., Schoenberg J. B. (1994). Second cancers following oral and pharyngeal cancers: role of tobacco and alcohol.. J Natl Cancer Inst.

[OCR_00832] Dolcetti R., Doglioni C., Maestro R., Gasparotto D., Barzan L., Pastore A., Romanelli M., Boiocchi M. (1992). p53 over-expression is an early event in the development of human squamous-cell carcinoma of the larynx: genetic and prognostic implications.. Int J Cancer.

[OCR_00851] Field J. K., Pavelic Z. P., Spandidos D. A., Stambrook P. J., Jones A. S., Gluckman J. L. (1993). The role of the p53 tumor suppressor gene in squamous cell carcinoma of the head and neck.. Arch Otolaryngol Head Neck Surg.

[OCR_00840] Field J. K., Spandidos D. A., Malliri A., Gosney J. R., Yiagnisis M., Stell P. M. (1991). Elevated P53 expression correlates with a history of heavy smoking in squamous cell carcinoma of the head and neck.. Br J Cancer.

[OCR_00843] Field J. K., Spandidos D. A., Stell P. M. (1992). Overexpression of p53 gene in head-and-neck cancer, linked with heavy smoking and drinking.. Lancet.

[OCR_00856] Franco E. L., Kowalski L. P., Kanda J. L. (1991). Risk factors for second cancers of the upper respiratory and digestive systems: a case-control study.. J Clin Epidemiol.

[OCR_00865] Harris C. C., Hollstein M. (1993). Clinical implications of the p53 tumor-suppressor gene.. N Engl J Med.

[OCR_00861] Harris C. C. (1993). p53: at the crossroads of molecular carcinogenesis and risk assessment.. Science.

[OCR_00866] Haughey B. H., Gates G. A., Arfken C. L., Harvey J. (1992). Meta-analysis of second malignant tumors in head and neck cancer: the case for an endoscopic screening protocol.. Ann Otol Rhinol Laryngol.

[OCR_00874] Hollstein M., Sidransky D., Vogelstein B., Harris C. C. (1991). p53 mutations in human cancers.. Science.

[OCR_00875] Hsu T. C., Johnston D. A., Cherry L. M., Ramkissoon D., Schantz S. P., Jessup J. M., Winn R. J., Shirley L., Furlong C. (1989). Sensitivity to genotoxic effects of bleomycin in humans: possible relationship to environmental carcinogenesis.. Int J Cancer.

[OCR_00882] Larson J. T., Adams G. L., Fattah H. A. (1990). Survival statistics for multiple primaries in head and neck cancer.. Otolaryngol Head Neck Surg.

[OCR_00889] Levine A. J. (1992). The p53 tumor-suppressor gene.. N Engl J Med.

[OCR_00891] Maestro R., Dolcetti R., Gasparotto D., Doglioni C., Pelucchi S., Barzan L., Grandi E., Boiocchi M. (1992). High frequency of p53 gene alterations associated with protein overexpression in human squamous cell carcinoma of the larynx.. Oncogene.

[OCR_00907] Nees M., Homann N., Discher H., Andl T., Enders C., Herold-Mende C., Schuhmann A., Bosch F. X. (1993). Expression of mutated p53 occurs in tumor-distant epithelia of head and neck cancer patients: a possible molecular basis for the development of multiple tumors.. Cancer Res.

[OCR_00898] Nigro J. M., Baker S. J., Preisinger A. C., Jessup J. M., Hostetter R., Cleary K., Bigner S. H., Davidson N., Baylin S., Devilee P. (1989). Mutations in the p53 gene occur in diverse human tumour types.. Nature.

[OCR_00912] Parshad R., Sanford K. K., Jones G. M. (1983). Chromatid damage after G2 phase x-irradiation of cells from cancer-prone individuals implicates deficiency in DNA repair.. Proc Natl Acad Sci U S A.

[OCR_00946] SLAUGHTER D. P., SOUTHWICK H. W., SMEJKAL W. (1953). Field cancerization in oral stratified squamous epithelium; clinical implications of multicentric origin.. Cancer.

[OCR_00918] Sakai E., Tsuchida N. (1992). Most human squamous cell carcinomas in the oral cavity contain mutated p53 tumor-suppressor genes.. Oncogene.

[OCR_00925] Schantz S. P., Hsu T. C. (1989). Mutagen-induced chromosome fragility within peripheral blood lymphocytes of head and neck cancer patients.. Head Neck.

[OCR_00930] Schantz S. P., Spitz M. R., Hsu T. C. (1990). Mutagen sensitivity in patients with head and neck cancers: a biologic marker for risk of multiple primary malignancies.. J Natl Cancer Inst.

[OCR_00942] Shikhani A. H., Matanoski G. M., Jones M. M., Kashima H. K., Johns M. E. (1986). Multiple primary malignancies in head and neck cancer.. Arch Otolaryngol Head Neck Surg.

[OCR_00937] Shin D. M., Kim J., Ro J. Y., Hittelman J., Roth J. A., Hong W. K., Hittelman W. N. (1994). Activation of p53 gene expression in premalignant lesions during head and neck tumorigenesis.. Cancer Res.

[OCR_00954] Spitz M. R., Fueger J. J., Beddingfield N. A., Annegers J. F., Hsu T. C., Newell G. R., Schantz S. P. (1989). Chromosome sensitivity to bleomycin-induced mutagenesis, an independent risk factor for upper aerodigestive tract cancers.. Cancer Res.

[OCR_00960] Vogelstein B., Kinzler K. W. (1992). p53 function and dysfunction.. Cell.

[OCR_00962] Wang L. D., Hong J. Y., Qiu S. L., Gao H., Yang C. S. (1993). Accumulation of p53 protein in human esophageal precancerous lesions: a possible early biomarker for carcinogenesis.. Cancer Res.

[OCR_00968] Watling D. L., Gown A. M., Coltrera M. D. (1992). Overexpression of p53 in head and neck cancer.. Head Neck.

[OCR_00971] Westra W. H., Offerhaus G. J., Goodman S. N., Slebos R. J., Polak M., Baas I. O., Rodenhuis S., Hruban R. H. (1993). Overexpression of the p53 tumor suppressor gene product in primary lung adenocarcinomas is associated with cigarette smoking.. Am J Surg Pathol.

